# Genetic Diversity of Sapovirus in Children, Australia

**DOI:** 10.3201/eid1201.050846

**Published:** 2006-01

**Authors:** Grant S. Hansman, Naokazu Takeda, Kazuhiko Katayama, Elise T.V. Tu, Christopher J. McIver, William D. Rawlinson, Peter A. White

**Affiliations:** * National Institute of Infectious Diseases, Tokyo, Japan;; † University of New South Wales, Sydney, New South Wales, Australia;; ‡ Prince of Wales Hospital, Sydney, New South Wales, Australia

**Keywords:** Sapovirus, genogroup, recombinant, infections, dispatch

## Abstract

Sapovirus was detected in 7 of 95 stool specimens from children with gastroenteritis of unknown etiology in Sydney, Australia, from August 2001 to August 2002 and from February 2004 to August 2004, by using reverse transcription–polymerase chain reaction. Sequence analysis of the N-terminal capsid region showed all human sapovirus genogroups.

Sapovirus (SaV), a member of the genus *Sapovirus* in the family *Caliciviridae*, is an etiologic agent of human gastroenteritis. SaV-associated infections can cause both mild and acute gastroenteritis. Symptoms include watery stool, mild and or acute diarrhea, stomach cramps, nausea, and vomiting (*1*). In a recent study, the independent risk factor for SaV gastroenteritis in children was contact with an index case-patient, usually in daycare centers (*2*). The most widely used method of SaV detection is reverse transcription–polymerase chain reaction (RT-PCR), which has high sensitivity and can be used for further genetic analysis (*3**,**4*). SaV strains can be divided into 5 genogroups (GI, GII, GIII, GIV, and GV), of which GI, GII, GIV, and GV strains infect humans, while GIII strains infect pigs (*5**,**6*). The 4 human genogroups can be further divided into genotypes (*7*). The purpose of this study was to describe sapovirus-associated infections in Australia.

## The Study

We screened stool specimens for SaV by using RT-PCR and described the genetic diversity of virus-positive specimens. A total of 95 stool specimens were collected from children <18 years of age treated for gastrointestinal illness at the Sydney Children's Hospital. Stool specimens were obtained from patients with gastroenteritis of unknown origin despite extensive investigation. These specimens were negative for common foodborne bacterial pathogens (*Salmonella*, *Shigella*, and *Campylobacter*) and enteric viruses (rotavirus, adenovirus, astrovirus, and norovirus) (IDEIA enzyme-linked immunosorbent assay, Dako Cytomation, Ely, UK). The specimens included 67 of 110 specimens obtained from children hospitalized between August 2001 and August 2002 and 28 of 60 specimens from outpatients between February 2004 and August 2004. Specimens were not tested for the presence of SaV if an etiologic agent was already identified (n = 75).

RNA was extracted and purified as described elsewhere (*3*). Ten microliters of RNA was reverse transcribed by using SuperScript III RNaseH (–) reverse transcriptase according to the manufacture's instructions (Invitrogen, Carlsbad, CA, USA). PCR was conducted by using a nested approach with primers directed against the N-terminal capsid region (*4*). The PCR products were analyzed by 2% agarose gel electrophoresis and visualized by staining with ethidium bromide. PCR-generated amplicons were excised from the gel and purified by using the QIAquick gel extraction kit (Qiagen, Hilden, Germany). Nucleotide sequences were determined by using the terminator cycle sequence kit (version 3.1) and the ABI 3100 Avant sequencer (Perkin-Elmer ABI, Boston, MA, USA). Nucleotide sequences were aligned by using ClustalX, and distances were calculated by using the 2-parameter method of Kimura (*8*). Phylogenetic trees with bootstrap analysis from 1,000 replicas were generated by using the neighbor-joining method as described previously (*8*).

SaV was detected in 7 of 95 stool specimens from children 9 months to 7 years of age with previously unknown causes of acute gastroenteritis. This represented a minimum prevalence of 4.1% (7 of 170 specimens). Sequence analysis showed the presence of all known human SaV genogroups (Figure). Two sequences (strains Sydney31 and Sydney40), which shared ≈99% nucleotide (nt) identity and belonged to genogroup GI, closely matched (>99% nt identity) the Manchester sequence. Three sequences (strains Sydney53, Sydney77, and Sydney4106) that belonged to genogroup GII had 69%–77% nt identity. Sydney4106 had ≈98% nt identity with the Mc10 sequence, Sydney53 had ≈90% nt identity with the C12 sequence, and Sydney77 had ≈99% nt identity with the Bristol sequence.

**Figure:**
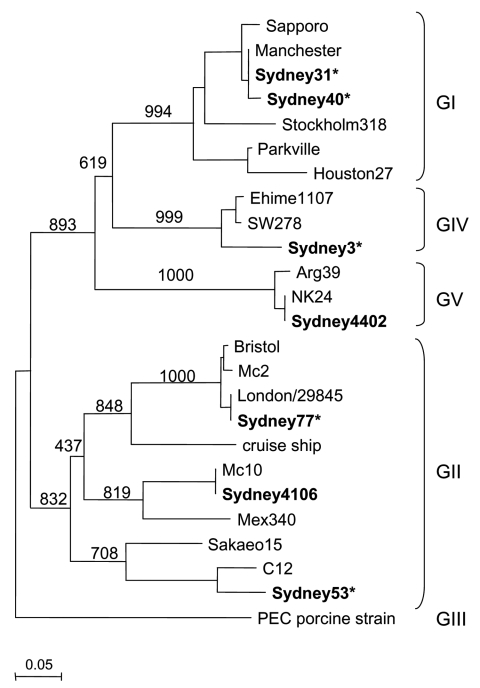
Phylogenic tree of Sapovirus (SaV) sequences isolated in this study (represented in **boldface**). SaV nucleotide sequences were constructed with the partial N-terminal capsid region, using SaV PEC strain (a porcine SaV) as an outgroup. The numbers on the branches indicate the bootstrap values for the clusters. Bootstrap values >950 were considered statistically significant for the grouping (*8*). Asterisks indicate specimens collected from outpatients. The distance scale in nucleotide substitutions per position is shown. Sydney isolates have been deposited in GenBank (accession nos. DQ104357–DQ104363). GenBank accession numbers for the reference strains are as follows: Arg39, AY289803; Bristol/98, AJ249939; C12, AY603425; cruise ship/2000, AY289804; Ehime1107, DQ058829; Houston/27/90, U95644; London/29845/90, U95645; Manchester, X86560; Mc2, AY237419; Mc10, AY237420; Mex340/1990, AF435812; NK24, AY646856; Parkville, U73124; PEC, AF182760; Sapporo/82, U65427; Stockholm/318/97, AF194182; Sakaeo15, AY646855; and SW278, DQ125333.

We recently reported SaV strains Mc10 and C12 as recombinant strains (*7*). Phylogenetic analysis of the nonstructural region (i.e., genome start to capsid start) grouped Mc10 and C12 in 1 GII cluster (*7*), and the structural region (i.e., capsid start to genome end) grouped Mc10 and C12 into distinct GII genotypes (*7*). Evidence suggested that the recombination site occurred at the polymerase and capsid junction in open reading frame 1, as we recently described with recombinant norovirus strains (*9*). Further sequence analysis of the nonstructural region (i.e., 800 nt of the polymerase gene) showed that Sydney4106 had ≈99% nt identity with Mc10, and Sydney53 had ≈92% nt identity with C12. These findings suggest that Sydney4106 and Sydney53 were also recombinant strains and indicate the widespread distribution and genetic stability of recombinant SaV strains. One sequence (strain Sydney3) belonged to genogroup GIV and had ≈99% nt identity with the SW278 sequence, which recently caused an outbreak of gastroenteritis in adults in Sweden in March 2004 (*1*). Another sequence (strain Sydney4402) belonged to genogroup GV and had 100% nt identity with the NK24 sequence, which was isolated from an infant with gastroenteritis in Thailand in December 2002 (*10*). White blood cells were detected in the stool specimens of 3 children infected with SaV genogroups GII, GIV, and GV (strains Sydney4106, Sydney3, and Sydney4402, respectively). In our previous study (*10*), an infant infected with NK24 (SaV genogroup GV) had a fever for 11 days and vomiting for 3 days, which was notably longer than the duration of symptoms in other infants infected with SaV GI and GII strains (unpub. data). These results suggest that some SaV genogroups could be more virulent than others, although additional studies are needed.

## Conclusions

Little is known about SaV infections in Australia (*11**–**14*). Data from these reports indicate that SaV is an uncommon cause of acute gastroenteritis in Australia. When the proportion of SaV present in the total calicivirus isolations was used, SaV was estimated to be the etiologic agent of gastroenteritis in 0.56% (*11*), 0.32% (*12*), and 0.46% (*14*) of cases. Our results have shown that SaV is an important cause of acute gastroenteritis in children in Sydney, with a minimum prevalence of 4.1%, which is higher than previously reported. This is the first report of SaV GIV genogroup-associated infection in Australia and widespread distribution of SaV. However, a more comprehensive study is needed to determine whether predominant SaV strains are circulating, as observed with noroviruses (*7**,**11**,**15*).
